# Material Removal Characteristics of Spherical-Array-Focused Ultrasonic Abrasive Machining

**DOI:** 10.3390/mi14020382

**Published:** 2023-02-03

**Authors:** Bo Du, Jinhu Wang, Julong Yuan, Binghai Lyu, Xinqian Zhang, Chunyu Zhang

**Affiliations:** 1 College of Mechanical Engineering, Zhejiang University of Technology, Hangzhou 310023, China; 2 Key Laboratory of Special Purpose Equipment and Advanced Processing Technology, Ministry of Education and Zhejiang Province, Zhejiang University of Technology, Hangzhou 310023, China; 3 Research Center of Laser Fusion, China Academy of Engineering Physics, Mianyang 621900, China

**Keywords:** focused ultrasound, ultrasonic abrasive machining, ultrasonic cavitation, quartz glass, material removal characteristics

## Abstract

To improve the ultrasonic energy and realize far-field ultrasonic abrasive machining of complex surfaces, a spherical-array-focused ultrasonic abrasive machining system was established. By combining ultrasonic field simulation, detection and a single-factor experiment, the influences of the ultrasonic generator current, abrasive concentration, and particle size on the material removal properties and surface quality evolution of quartz glass were investigated. When the current was less than 0.4 A, the material removal showed plastic removal at the nanoscale. When the current was more than 0.5 A, the cavitation phenomenon formed micron-scale impact removal traces on the workpiece surface. The increase in abrasive concentration increased the impact density and material removal rate, while excessive abrasive concentration increased the impeding effect between abrasive particles and reduced the material removal rate. Moreover, the increase in abrasive particle concentration enhanced heterogeneous cavitation nucleation, promoted the removal of abrasive impact materials under the action of a cavitation jet, and inhibited the removal of direct surface cavitation. The abrasive particle size affects the heterogeneous cavitation nucleation and the acceleration of the cavitation jet on abrasive particles, which affects the material removal rate and surface quality. By controlling the energy of the focused ultrasound and abrasive parameters, the plastic or brittle domain removal of quartz glass can be achieved at the micro- and nanoscales.

## 1. Introduction

Ultrasonic machining technology is widely used in the field of ultra-precision machining of difficult-to-machine materials [1,2,3]. A series of ultrasonic-assisted machining technology has been developed by combining ultrasonic technology with cutting, grinding, and other traditional processing technologies [4,5,6]. With the development of special processing technologies such as electronic processing, plasma processing and laser processing, many more advanced composite processing technologies have been developed by the combination of ultrasonic and various special processing technologies [7]. Benefiting from this, problems such as low efficiency and poor surface quality existing in traditional processing methods have been effectively improved [8,9,10]. The application range of ultrasonic technology is constantly expanding, and it has become an important means in the field of high-tech processing [11]. Non-contact ultrasonic abrasive machining is a variant of ultrasonic machining, in which the material is removed predominantly by cavitation erosion, abrasive particle micro-cutting, or micro-plowing in an abrasive slurry [12,13,14]. However, further research is still required to realize the controllable removal of difficult-to-process materials by exploiting the advantages of good penetration, strong beam directivity, and flexible control of ultrasonic waves.

Many studies have been conducted on ultrasonic-assisted abrasive processing technology. Ichida et al. [15] determined the removal effects of abrasive particles on a workpiece under ultrasonic action through comparative experiments and believed that the impact and scratching of abrasive particles on the workpiece surface under the action of ultrasonic vibration can achieve nanoscale removal. Ralchenko et al. [16] processed polycrystalline CVD diamond films using ultrasound, which reduced the surface roughness by an order of magnitude in a few minutes. Beaucamp et al. [17] used an acoustic lens and conical cavity to make the focus of the sound wave coincide with the outlet of the abrasive flow nozzle, forming an ultrasonic composite jet acting on the workpiece surface. The material removal rate increased by 380% compared with traditional fluid processing. Qi et al. [18] proposed an ultrasonic vibration suspension polishing method, which uses a standing-wave ultrasonic field to form the high and low ultrasonic pressure region, induces abrasive impact in the polishing fluid to achieve material removal, and performs polishing experiments on tungsten steel, effectively reducing the workpiece surface roughness and reducing the workpiece surface residual stress. Yu et al. [19] proposed an ultra-smooth surface-processing method based on two-dimensional fluid vibration. Ultrasonic vibration was applied to the fluid in two directions to form a stable ultrasonic field. Under the action of an ultrasonic field, the abrasive impacts the workpiece surface at a certain speed to achieve material removal. Li et al. [20] replaced the ultrasonic generating device in the traditional ultrasonic-assisted abrasive polishing method with a transducer array and used multiple transducers to form an acoustic pressure region to drive the abrasive flow carrying abrasive particles to impact the workpiece surface, thus realizing nanoscale material removal.

Current ultrasonic abrasive machining methods are primarily based on two principles. One is that there is a certain gap between the vibration tool and workpiece surface, and the abrasive medium fills the gap. Ultrasonic vibration and cavitation are used to stimulate abrasive impact on the workpiece surface for processing. This method has a large amount of removal; however, the surface quality of the workpiece is poor, and machining action occurs in the near field of the tool head; therefore, it is not suitable for machining complex surfaces. The other is to form a regional ultrasonic field in the abrasive medium through different transducer array arrangements and use the regional ultrasonic pressure to drive the abrasive impact on the workpiece surface to achieve the removal. The ultrasonic pressure generated by this method is relatively dispersed, and the energy is limited; therefore, it is difficult to achieve effective material removal. At present, little research is being carried out on far-field ultrasonic abrasive machining. If the ultrasonic energy can be concentrated far away from the ultrasonic source to drive the abrasive particles in the slurry for machining, a complex surface can be machined by avoiding interference of tool and workpiece.

To increase the energy of the focused ultrasonic field and realize far-field ultrasonic abrasive machining, a spherical-array-focused ultrasonic abrasive machining system was established. Combined with ultrasonic field simulation, ultrasonic field detection and a single-factor experiment, the influences of the ultrasonic generator current, abrasive concentration, and particle size on the material removal properties and surface quality evolution of quartz glass were investigated.

## 2. Experiment

### 2.1. Experimental Platform

**Figure 1:**
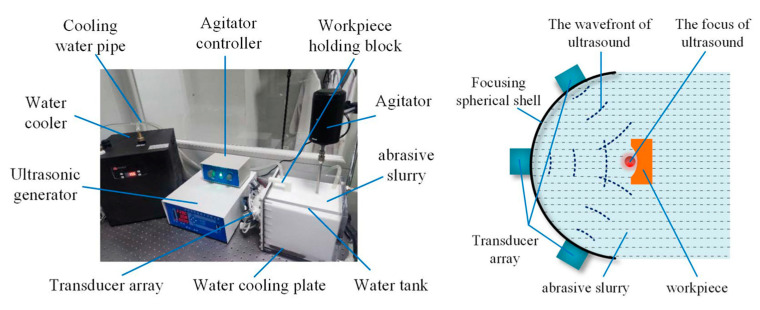
Experimental platform and machining principle of spherical-array-focused ultrasonic abrasive machining.

Figure 1 shows the spherical-array-focused ultrasonic abrasive machining system, including an ultrasonic generator (HX-F1-1500), spherical array of 28 kHz transducer, industrial chiller (ALGY-III), water cooling plate, and gripper. Ultrasonic field detection was conducted using a probe hydrophone (NH4000) and oscilloscope. Five transducers were distributed on the spherical shell in two vertical directions to form a spherical array. During machining, the ultrasonic generator sends ultrasonic signals to stimulate the transducer array element to produce mechanical vibrations. The vibration generated by each array element forms a wavefront that propagates forward in the abrasive slurry medium, and each wavefront forms a high-intensity ultrasonic pressure gathering area at the focusing center. The quartz glass workpiece is fixed at the focusing center, and the abrasive particles in the slurry impact the workpiece by the action of concentrated acoustic pressure to remove the surface material.

### 2.2. Focused Ultrasonic Field Simulation and Detection

COMSOL software was used to simulate the characteristics of the focused ultrasonic field. In the experimental platform, the transducer was arranged symmetrically in the two directions of the spherical shell. To simplify the model, a two-dimensional cross-section in one direction was simulated and analyzed. The transient pressure acoustic physical field was selected as the simulated physical field, and the domain equation is the ultrasonic pressure wave equation:

(1) $$\frac{1}{\rho c^{2}}\frac{\partial^{2}P_{t}}{\partial t^{2}}+\nabla \times \left(-\frac{1}{\rho }\right)\left(\nabla P_{t}-q_{d}\right)=Q_{m}$$

where ρ, *c*, *P_t_*, *q_d_*, and *Q_m_* are the medium density, speed of sound, absolute acoustic pressure, dipole source, and unipolar source, respectively.

**Figure 2:**
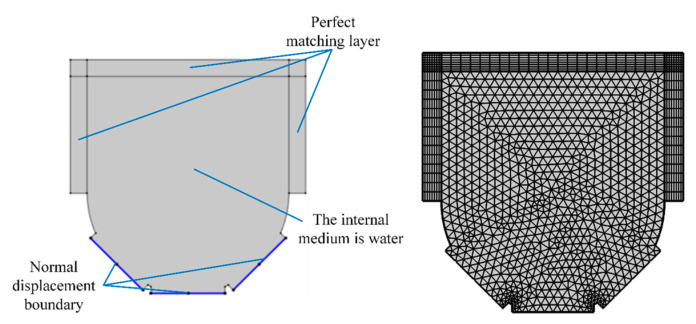
Physical model of simulation.

The physical simulation model is illustrated in Figure 2. In the simulation, the transducer interface is defined by the normal displacement, which is defined by a sine function of the unit amplitude. The internal medium was set as water, and the acoustic impedance of the external spherical shell was greater than that of the internal medium; therefore, the rest of the boundaries were set as hard sound field boundaries. A large slurry container was connected to the front of the shell, thus creating perfectly matched layers in the top three directions to absorb sound waves. The maximum mesh size was less than 1/6th of the wavelength to ensure the accuracy of the simulation. A triangular mesh shape was selected, and the distribution layer was set as the perfect matching layer. The other simulation parameters are presented in Table 1.

**Figure 3:**
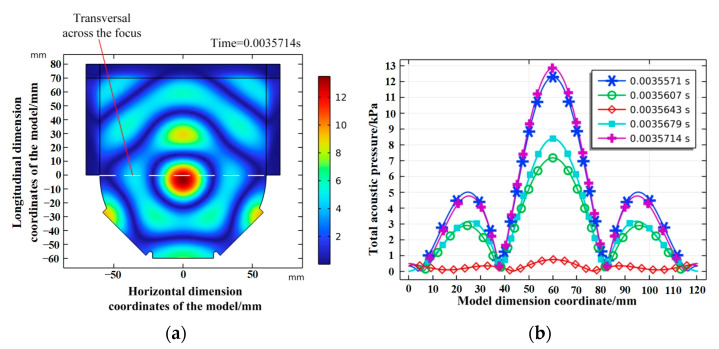
The pressure distribution of the focused ultrasonic field. (**a**) Stable focus-moment ultrasonic field. (**b**) Acoustic pressure distribution across focal transversal.

The time step was set to 1/10th of the period. The simulation results show evident ultrasonic pressure accumulation, and the ultrasonic field at the stable focusing moment is shown in Figure 3a. Furthermore, after forming the ultrasonic pressure focus, the ultrasonic pressure value and shape of the ultrasonic field show periodic changes, and each cycle contains five time steps. Take a single cycle period after ultrasonic field stabilization, and the ultrasonic pressure distribution at five moments passing through the focal point transection is shown in Figure 3b. When all moments are superimposed, the ultrasonic field presents a focused state on the whole, and the concentrated acoustic pressure fluctuates.

To detect the distribution of acoustic pressure, probe hydrophones and oscilloscopes were used to detect the transverse acoustic pressure passing through the focus center of the acoustic field. The oscilloscope detection value was the effective voltage value, and the acoustic pressure value is calculated using the acoustic pressure calculation equation:

(2) $$P=10^{PL/20}\times P(\mathrm{ref})$$

where *P* is acoustic pressure, *PL* is the acoustic pressure level, and *P*(ref) is the reference acoustic pressure, which is 1 × 10^−6^ Pa in underwater sound; the calculation formula of *PL* is:

(3) $$PL=20\log_{10}\left[U_{0}\right]-M$$

where *U*_0_ is the effective voltage value detected by the oscilloscope, *M* is the sensitivity of the hydrophone, which was calibrated before use, and the sensitivity at 28 kHz is −221.2 dB. The acoustic pressure value can be calculated by combining Equations (2) and (3).

**Figure 4:**
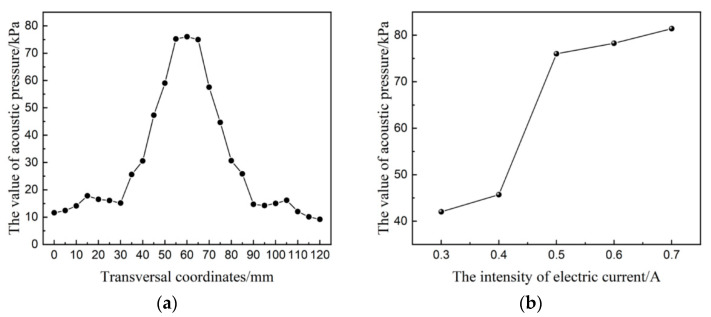
Acoustic pressure detection curve. (**a**) Through focal point transversal. (**b**) Focal point acoustic pressure curve with current.

When the current was 0.5 A, the detection curve of acoustic pressure distribution through the transversal of the focusing center is shown in Figure 4a. The detection result also shows evident acoustic pressure accumulation; however, compared with the simulation result, the detection value of the acoustic pressure in the focusing center is relatively larger. During the experiment, the acoustic pressure of the focusing center was controlled by adjusting the current of the ultrasonic generator. The minimum current was set as 0.3 A and the maximum current as 0.7 A. A probe hydrophone was used to detect the acoustic pressure of the focusing center under each current, and the test results are shown in Figure 4b. The acoustic pressure value increased with increasing current. When the current value was 0.3–0.4 A, the acoustic pressure was weak. When the current value was 0.5 A, the acoustic pressure value of the focusing center increased sharply.

When ultrasonic waves were applied to an abrasive slurry, ultrasonic cavitation resulted in the growth, oscillation, and bursting of bubbles in the liquid. The minimum acoustic pressure required to trigger cavitation is the cavitation threshold. Ultrasonic cavitation generates pressure and temperature fields in the local region of the slurry [21]. The transducer frequency used in the experimental platform was 28 kHz. When the current was 0.5 A, the maximum acoustic pressure in the focusing center area increased sharply, reaching 76 kPa. Ultrasonic cavitation is believed to occur in the slurry, leading to a sudden change in the local pressure. As the current continued to increase, cavitation was enhanced, and the acoustic pressure value in the focusing region also increased.

### 2.3. Experimental Process

Single-factor experiments were conducted to investigate the influence of the ultrasonic generator current, abrasive particle size, and abrasive particle concentration on the material removal characteristics of quartz glass. Considering the high hardness of quartz glass, alumina abrasive with a Moh hardness of 9 and crystalline phase of α was selected to prepare the slurry. The other experimental conditions are listed in Table 2.

In the experiment, a precision balance (SARTOURIUS MSE225S-10E-DU) was used to measure the removal weight thrice and calculate the mean value. The surface roughness was detected using a profiler (Form TALYSUR I60). A Gaussian filter is selected for measuring the surface roughness in the machining area. The average surface roughness was calculated using three sampling lines with a length of 1.5 mm. The surface morphology of the workpiece was detected using a laser interferometer (Zygo), microscope (KEYENCE VHX-6000), atomic force microscope (Dimension Icon), and white-light interferometer (SuperView W1).

## 3. Experimental Results and Analysis

### 3.1. Effect of Current Change

**Figure 5:**
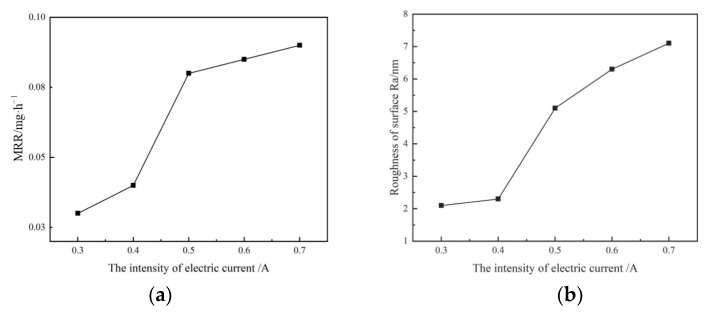
Curves of the material removal rate and roughness with the current intensity. (**a**) Curve of material removal r. (**b**) Curve of surface roughness.

The abrasive slurry is a mixture of alumina abrasive and deionized water. The alumina abrasive has a particle size of 2.5 μm and mass concentration of 10%. The quartz glass workpiece was processed under different currents, and the material removal rate (MRR) and surface roughness *R*_a_ are shown in Figure 5. Based on the experimental results, when the ultrasonic generator current was 0.3 and 0.4 A, the material removal rate was small, and *R*_a_ in the machining area was basically unchanged. When the current was increased from 0.5 to 0.7 A, the material removal rate and *R*_a_ increased sharply. According to the test results of the ultrasonic field, as shown in Figure 4b, this mutation is related to the mutation of acoustic pressure and the generation of the cavitation phenomenon.

**Figure 6:**
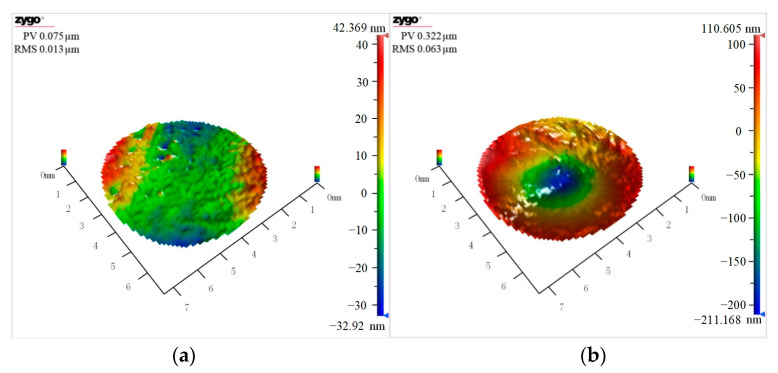
Topography contrast diagram. (**a**) Original morphology of workpiece. (**b**) Workpiece morphology after 0.5A current processing.

A laser interferometer was used to observe the overall removal topography, and surface topography changes in the machining area were observed under high-current machining. A comparison between the original surface and surface removal topography under 0.5 A current machining is shown in Figure 6. The machining area exhibited circular pits with a depth of approximately 322 nm. The material under high acoustic pressure in the focusing center was removed the most, while the material in the areas far away from the focusing center was less removed. The focusing ultrasonic field achieved a fixed-point removal on the workpiece surface.

**Figure 7:**
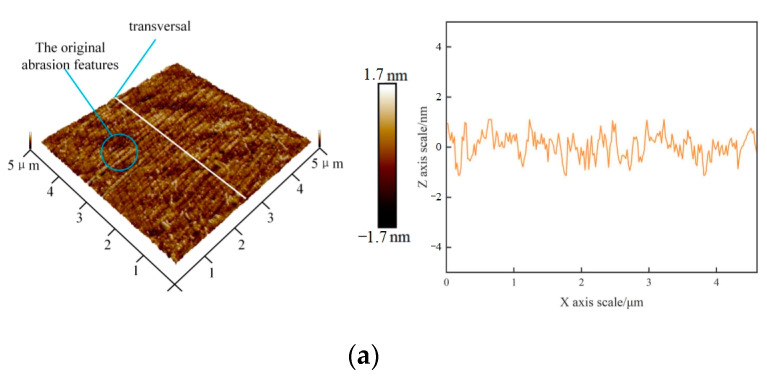

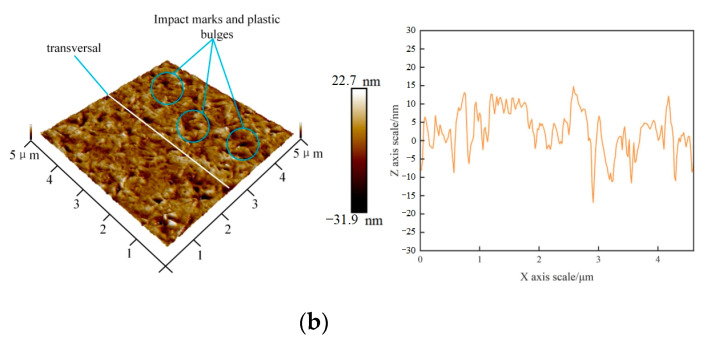
Comparison of microscopic morphology. (**a**) Original morphology and cross-section profile of the workpiece. (**b**) Workpiece morphology and cross-section profile after 0.3 A current processing.

No evident macro-removal morphology on the workpiece surface under 0.3 A and 0.4 A current machining occurred; however, the change in weight removal shown in Figure 5 indicates that small-scale removal occurred on the workpiece surface. To further characterize such small-scale removal, atomic force microscopy was used to observe the surface morphology of the workpiece before and after processing with a current of 0.3 A. The surface morphology and cross-section profile of the workpiece are shown in Figure 7. The original abrasion features of the workpiece surface were completely removed and replaced with some plastic machining marks, indicating a nanoscale plastic removal of the workpiece surface at a small current. Combined with the ultrasonic field test results shown in Figure 4b, cavitation had not occurred in this case, and it can be concluded that the removal effect was caused by the impact of abrasive particles on the workpiece surface under ultrasonic vibration excitation.

According to the ultrasonic theory, the acoustic pressure *P* at a point in the ultrasonic field can be expressed as

(4) P=ρνc

where ρ is the medium density of the abrasive slurry, *c* is the propagation velocity of the ultrasonic waves in the abrasive slurry medium, and *ν* is the particle vibration velocity in the abrasive slurry medium. In the case of a small current, the particle vibration velocity can be regarded as the impact velocity of abrasive particles, and it can be observed that the abrasive vibration velocity is positively correlated with the acoustic pressure. The material removal model of a single abrasive particle based on Hertz theory is used to describe the material removal volume of a single abrasive particle [20]:

(5) $$V=\pi R\delta^{2}-\frac{\pi \delta^{3}}{3}\approx \frac{0.86\pi \rho_{0}\nu^{2}R^{3}}{\sigma_{S}}$$

where *δ* is the pressing depth, *ρ*_0_ is the abrasive density, *ν* is the abrasive vibration velocity, *σ*_s_ is the material yield limit, and *R* is the contact ball radius. Equation (5) can be regarded as the removal model of abrasive particles impacting the workpiece surface under ultrasonic vibration excitation. Under the condition of a small current, the material removal rate and workpiece surface roughness changes were small; thus, plastic removal at the nanometer level could be realized. As the current increased, the focusing acoustic pressure and abrasive vibration velocity increased, and the removal effect was also enhanced.

**Figure 8:**
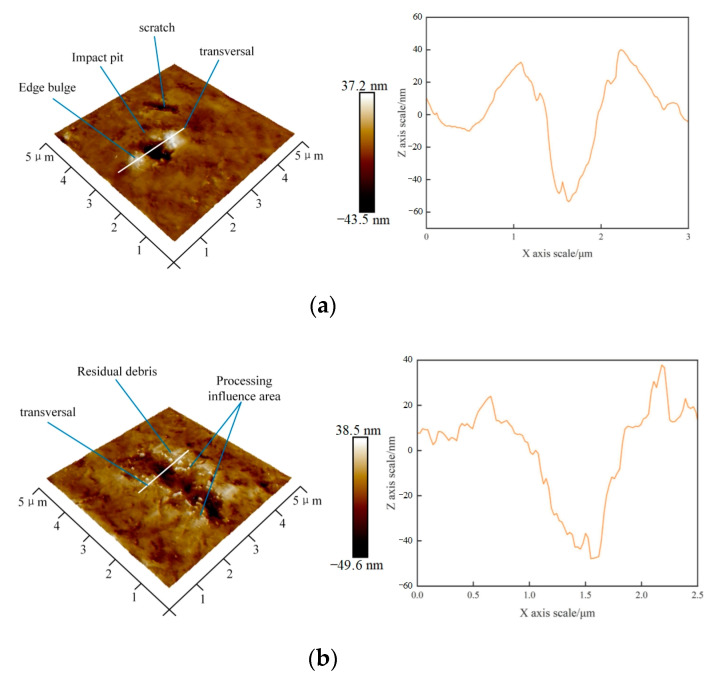
Micromorphology after 0.7 A current machining. (**a**) Small pit and pit section profile. (**b**) Large pit and pit section profile.

An atomic force microscope was used to observe the microscopic machining morphology under the 0.7 A processing condition. To eliminate the effect of the superposition of the removal marks as far as possible, an atomic force microscope was used to observe the area with sparse removal marks at the edge of the machining area. The workpiece surface morphology and local section profile of the machining marks are shown in Figure 8. Cavitation occurred in the area of acoustic pressure accumulation.

In Figure 8, two new typical removal features can be observed. The first is the pitted or scratched micron-scale removal marks with plastic protrusion around it, and the trace area is relatively small. Based on the topography and size of the pits, such processing marks may be formed by impact-scratching of abrasive particles driven by the cavitation jet generated by the cavitation phenomenon in the acoustic pressure accumulation area. The second type of processing mark is a pit surrounded by residual debris. Compared to the first type of processing mark, this type of mark has a larger impact area and stronger material removal ability. It can be concluded that the removal marks were caused by the direct action of the cavitation jet on the workpiece surface. With an increase in the current, cavitation occurs and continues to strengthen, which enhances the material removal effect, but further leads to an increase in surface roughness. The removal of abrasive particles that impact the workpiece surface under the excitation of ultrasonic vibration is further strengthened and synergistic with ultrasonic cavitation simultaneously.

When the current is small and no cavitation phenomenon occurs in the focusing area, the plastic removal of nano-scale material surface can be realized. However, the material removal is relatively weak: the removal mechanism is the abrasive particles impacting the workpiece surface under the excitation of ultrasonic vibration. When the current is higher, ultrasonic cavitation contributes to the removal effect, and the removal traces of the material show a greater removal effect. To further study the removal effect of a large current, the current was set at 0.7 A, and the removal effect was compared by changing the abrasive particle concentration and size. The removal-effect changes by a current of 0.3 A were taken as a reference to further study the removal characteristics of large currents.

### 3.2. Effect of Abrasive Particle Concentration Change

**Figure 9:**
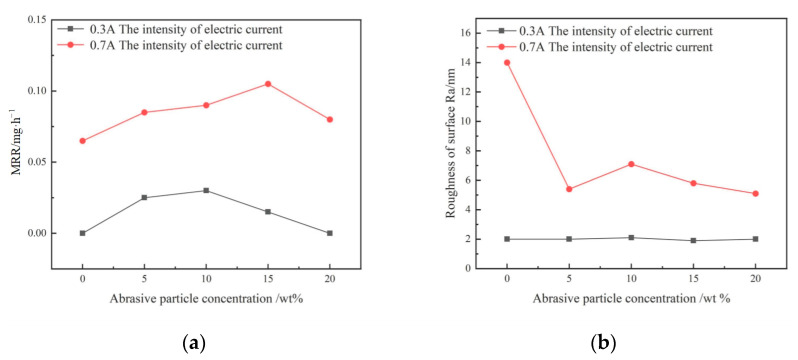
Curves of the material removal rate and roughness with the abrasive particle concentration. (**a**) Curve of material removal rate. (**b**) Curve of surface roughness.

An abrasive slurry with an alumina particle size of 2.5 μm and different mass concentrations was used to machine a quartz glass workpiece. The MRR and surface roughness *R*_a_ are shown in Figure 9. Based on the experimental results, when the current was 0.3 A, the material removal rate changed a little with the concentration. Particularly, when the abrasive particle concentration was 0% or reached 20%, there was no material removal and the surface roughness *R*_a_ in the machining area was unchanged. When the current was 0.7 A, the material removal rate increased with an increase in the abrasive concentration and then decreased. The machined surface roughness *R*_a_ was larger when the abrasive concentration was 0%; however, the *R*_a_ values at other concentrations were relatively low.

**Figure 10:**
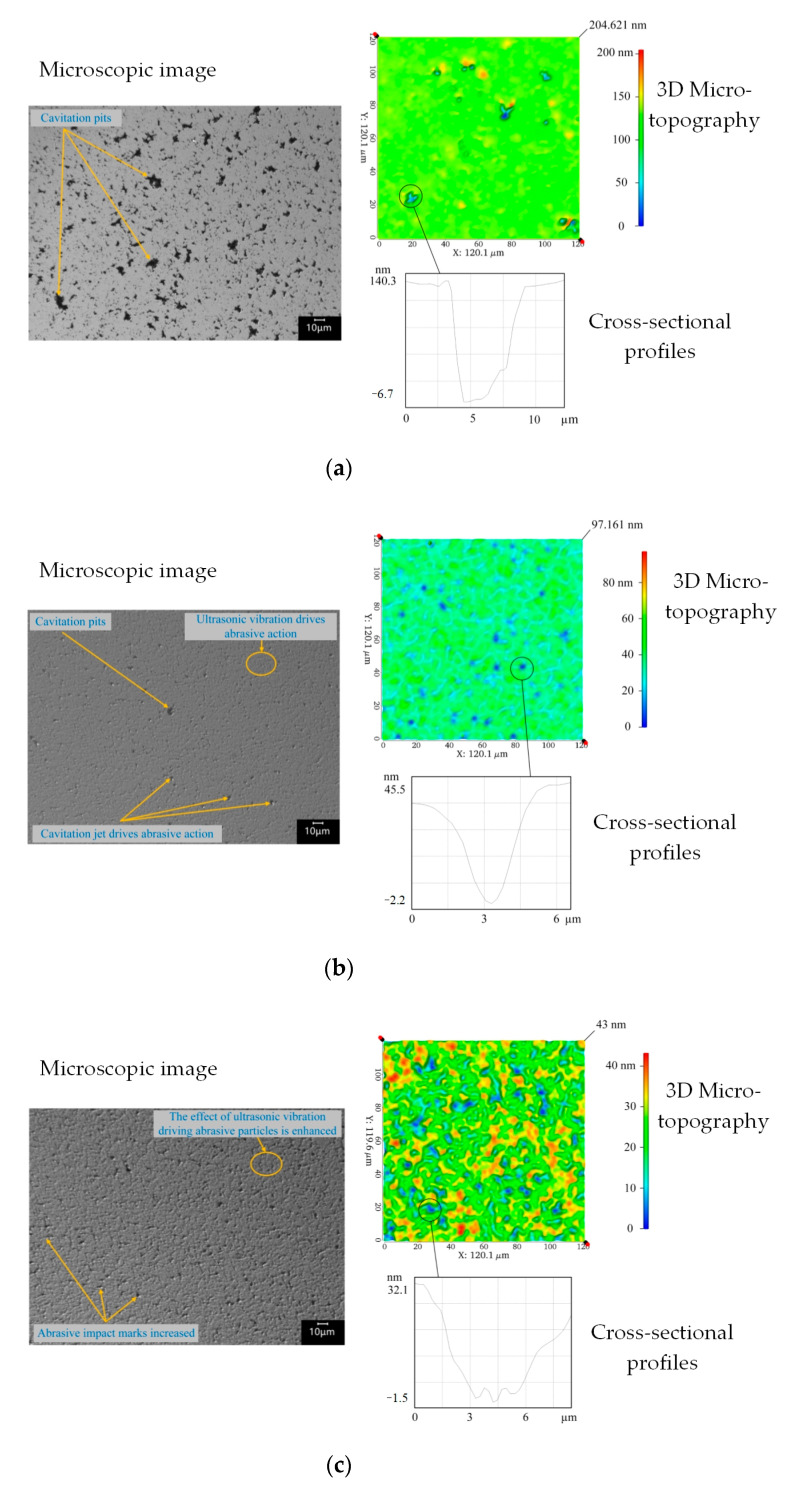
Comparison of machining morphology at different abrasive concentrations at a current of 0.7 A. (**a**) Abrasive particle concentration was 0%. (**b**) Abrasive particle concentration was 5%. (**c**) Abrasive particle concentration was 15%.

When the abrasive particle concentration was 0%, no material was removed under 0.3 A current, and the workpiece morphology was the same as the original morphology. Material removal under 0.7 A current was evident. A comparison of the machining morphology for several abrasive particle concentrations is shown in Figure 10. Each picture was divided into two parts. The left side showed the microscopic image of the machining area, and the right side shows the 3D micro-topography and the cross-sectional profiles of a single pit. In Figure 10a, there is only a cavitation phenomenon but no abrasive action in the focusing center area, and the machining morphology consists of pits of different sizes, which are generated by the direct action of the cavitation jet on the workpiece surface. After the formation of these irregular pits, it is more conducive to cavitation nucleation to form further damage, and pits are further expanded, which also leads to a relatively higher roughness *R*_a_ in the processing area.

From the perspective of the overall morphology of the workpiece, when the current was 0.7A and the abrasive particle concentration was 0%, the number of large pits generated by the direct action of the cavitation jet was greater. The addition of abrasive particles led to heterogeneous cavitation nucleation; consequently, the number of large cavitation pits began to decrease, and cavitation occurred at the abrasive particles [22], inhibiting the direct removal of cavitation jets on the workpiece surface and the abrasive particles begin to impact the workpiece surface under the action of cavitation jets to produce material removal.

From the perspective of the morphology of a single pit, the depth of the cavitation pits on the workpiece surface was greater, and the edge was steeper when there were no abrasive particles. The cavitation pits formed on the workpiece surface after the addition of abrasive particles were significantly smaller than those formed without abrasive particles, and the edges of these pits were gentler. Some of these pits may have been formed after the formation of cavitation pits by the impact of cavitation jet-driven abrasive particles and ultrasonic vibration-driven abrasive particles, while the others may have been formed by the local crushing and expansion of workpiece surface materials under the impact of abrasive particles. The sizes of the pits also support this conclusion. Additionally, at other locations, the workpiece surface morphology exhibited smaller impact marks. Similar to the number of pits, these impact marks also became denser with an increase in the abrasive concentration. These removal features were formed by the action of the ultrasonic vibration-driven abrasives.

At a current of 0.3 A, no material removal without abrasive particles occurred, which further proves that at a current of 0.3 A, material removal is caused by abrasive particles impacting the workpiece surface under ultrasonic vibration. This small-scale removal effect has been analyzed in the previous section. When the current was 0.7 A, the acoustic pressure of the focusing center exceeded the cavitation threshold of the abrasive slurry, and the cavitation effect on the workpiece surface began to occur. Even in the absence of abrasive particles, the cavitation jet was directly removed. However, after the addition of abrasive particles, the direct removal of the cavitation jet was inhibited, and the removal effect of abrasive particles impacting the workpiece surface under the action of the cavitation jet began to occur. This also explains the two new removal traces shown in Figure 8.

When the current was 0.7 A, the overall removal effect was first enhanced with the increase in abrasive particle concentration. This is because abrasive particles impact the workpiece surface under the action of the cavitation jet and start material removal. The processing traces generated by this removal effect were relatively sparse when the abrasive particle concentration was 5%, indicating that the abrasive impact probability was small and the removal effect was weak at low concentrations. When the abrasive concentration was 15%, the processing marks were denser, indicating that the abrasive impact probability increased with increasing abrasive concentration, and the material removal rate correspondingly increased. However, with the increase in abrasive impact probability, the removal effect of abrasive impact on the workpiece surface under the action of ultrasonic vibration was also enhanced, which was proven by the intensive plowing and scratching marks on the workpiece topography. With an increase in the particle concentration, the hindrance between particles became stronger, and the overall removal effect decreased.

When the current was 0.3 A, the acoustic pressure of the focusing center was small, the driving force of the abrasive particles was weak, and the removal effect was more easily affected by the change in concentration. When the abrasive concentration was 0%, the acoustic pressure was not sufficient to produce a removal effect on the workpiece surface. With the increase in abrasive concentration, abrasive particles began to impact and scratched the workpiece surface under the action of ultrasonic vibration to form a removal effect, which was enhanced with the increase in abrasive concentration. When the abrasive concentration continued to increase, the removal effect weakened owing to the obstruction effect between the abrasive particles. This removal effect was affected by abrasive obstruction at concentrations lower than 0.7 A.

### 3.3. Effect of Abrasive Particle Size Change

**Figure 11:**
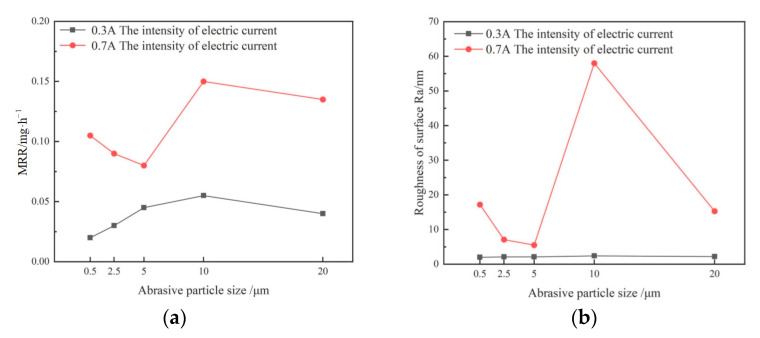
Curves of the material removal rate and roughness with the abrasive particle size. (**a**) Curve of material removal rate. (**b**) Curve of surface roughness.

Alumina abrasive slurry with a concentration of 10% was prepared with different abrasive particle sizes, and the quartz glass workpiece was processed with different abrasive particle sizes. The change curves of MRR and *R*_a_ with the abrasive particle size were shown in Figure 11. When the current was 0.3 A, the MRR at 10 μm particle size was relatively high, while the MRR at other particle sizes was low, and the *R*_a_ of the processing area showed no evident change. When the current was 0.7 A, the MRR fluctuated significantly. When the particle size was 10 μm, the MRR reached a maximum, and the *R*_a_ of the processing area suddenly increased.

**Figure 12:**
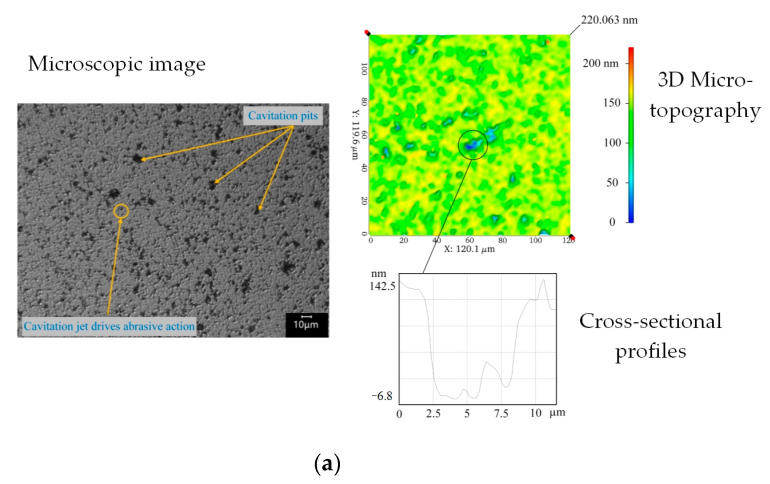

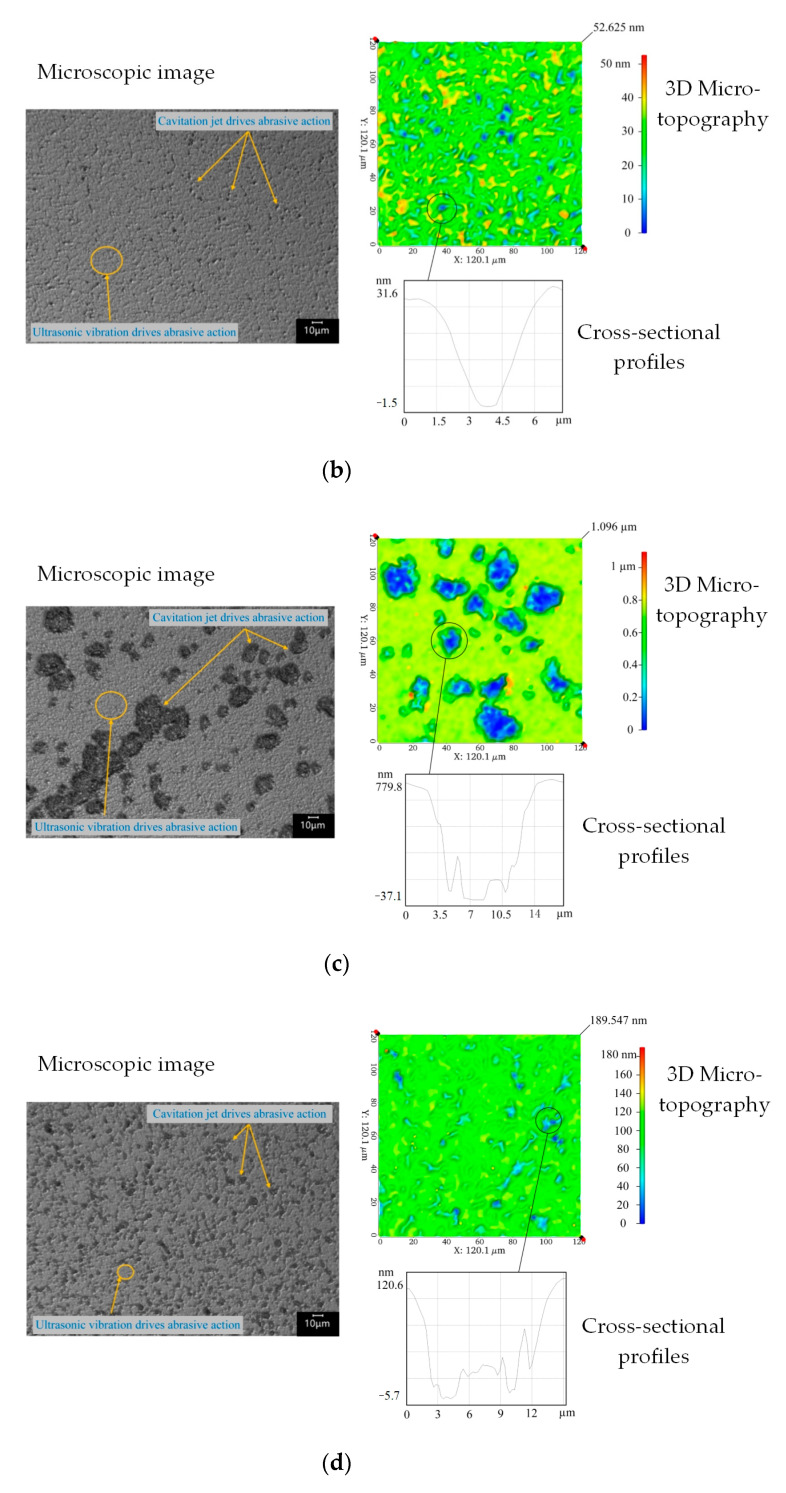
Comparison of machining morphology under different abrasive particle size. (**a**) Abrasive particle size was 0.5 μm. (**b**) Abrasive particle size was 2.5 μm. (**c**) Abrasive particle size was 10 μm. (**d**) Abrasive particle size was 20 μm.

Figure 12 shows a comparison of the machining morphology of several abrasive particle sizes when the current was 0.7 A. Numerous cavitation pits similar to those in Figure 10a were observed in the machining morphology when the abrasive particle size was 0.5 μm; however, the number of large cavitation pits and size of the cavitation pits were not significantly affected by the abrasive particles, and with a further increase in the abrasive particle size, the pits formed by direct cavitation gradually decreased. This is because the large size of the abrasive particles is more conducive to anisotropic cavitation nucleation and inhibits cavitation on the workpiece surface to a greater extent.

The surface morphology of the workpiece was highly sensitive to the particle size. When the particle size increased to 10 μm, the workpiece surface exhibited completely different morphologies. Similar large-sized cavitation pits in Figure 12a disappeared, and the newly formed pits on the workpiece surface were deeper. Their sizes were larger than those of the cavitation pits and close to those of the abrasive particles, which led to abrupt changes in the removal amount and roughness at this time. From the perspective of material removal, removal pits are formed by the impact of abrasive particles driven by cavitation jets. The pits are close to the theoretical analysis of single abrasive impact pits in the literature [23]. The sizes of pits formed by single or insufficient impacts are small, whereas the sizes of pits formed by the full impact of abrasive particles and multiple impacts are large. The removal of abrasive particles driven by ultrasonic vibration is achieved by abrasive vibration on the workpiece surface, which forms relatively flat processing marks in positions other than pits.

**Figure 13:**
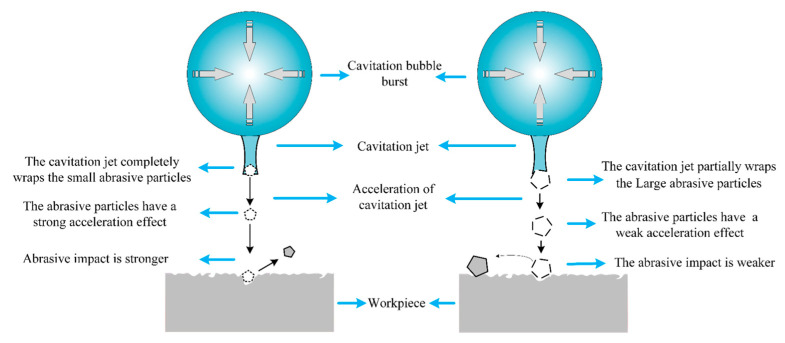
Diagram of cavitation jets acting on abrasive particles of different sizes.

Moreover, when the particle size increased to 20 μm, the workpiece surface did not form pits close to the particle size; however, a large number of irregular impact marks close to the depth of the cavitation pits were formed. The driving effect of a cavitating jet on abrasive particles was influenced by the size of the abrasive particles [22], and abrasive particles smaller than those of the jet particles realized significant acceleration under the coverage of the cavitating jet. However, when the particle size was larger, only a part of the particle was covered by the cavitation jet, and the particle became an obstacle between the cavitation jet and the workpiece surface. The depth of the abrasive particles on the workpiece surface was limited, and the mechanism is shown in Figure 13. Therefore, the irregular edge of the large-sized abrasive particle impinged on the workpiece surface under relatively weak acceleration, forming machining marks, as shown in Figure 12d. Considering the particle size and machining morphology of the abrasive particles, the size of the cavitation jet may range from 10 to 20 μm under the above experimental conditions, which is close to the theoretical analysis of the size of the cavitation jet in the literature [24].

## 4. Conclusions

In this study, a spherical-array-focused ultrasonic abrasive machining method was used to perform single-point removal processing of smooth quartz glass, and the material removal, roughness, and surface morphology under different processing parameters were tested and analyzed. The ultrasonic pressure at the focal point increased with increasing current, and ultrasonic cavitation occurred when the current reached 0.5 A. Analysis of experimental results under different parameters shows that:

(1) Cavitation does not occur in the focusing center of the ultrasonic field under a small current, and material removal is caused by the impact of abrasive particles on the workpiece surface driven by ultrasonic vibration. In this case, plastic material removal at the nanometer level can be achieved. With the increase of drive current, cavitation occurs and material removal is the collaborative removal caused by abrasive impacts on the workpiece surface under the excitation of ultrasonic vibration, ultrasonic cavitation jets directly acting on the workpiece surface, and abrasives impacting on the workpiece surface under the action of cavitation jet.
(2) The material removal rate increased firstly and then decreased with the increase of abrasive concentration. The heterogeneous cavitation–nucleation phenomenon generated by the addition of abrasive particles inhibited the intensity of direct cavitation action on the workpiece surface. The abrasive particle concentration affects the abrasive impact density, and material removal resulting from the ultrasonic cavitation jet directly acting on the workpiece surface is weakened.
(3) The material removal rate fluctuated with the change in the abrasive particle size. The abrasive particle size affects the intensity of the direct cavitation on the workpiece surface. A large abrasive size is more conducive to heterogeneous cavitation nucleation and has a stronger inhibitory effect on direct cavitation on the workpiece surface. More cavitation occurs at the position of abrasive particles, and the material removal resulting from abrasive particles impacting the workpiece surface under the action of the cavitation jet is enhanced. Moreover, the abrasive particle size affects the driving effect of the cavitation jet on the abrasive particles. Abrasive particles with smaller sizes are completely wrapped by the cavitation jet, which realized a stronger driving effect. However, abrasive particles with a larger size is partially wrapped by the cavitation jet, which became obstacles between the cavitation jet and workpiece surface.

Further research will focus on improving the ultrasonic energy and the focusing resolution to increase the machining efficiency and precision. By controlling ultrasonic deflection and focusing through a phased array, more flexible focusing control can be achieved.

## Figures and Tables

**Table 1:** Main parameters of simulation.

Parameters	Values
Spherical shell diameter (mm)	60
Diameter of transducer (mm)	44
Transducer arrangement angle (°)	45
Frequency (kHz)	28
Temperature (K)	293.15

**Table 2:** Experimental conditions.

Experimental Parameters	Parameter Value
Frequency (kHz)	28
Current intensity (A)	0.3, 0.4, 0.5, 0.6, 0.7
Abrasive size (μm)	0.5, 2.5, 5, 10, 20
Abrasive particle concentration (wt.%)	0, 5, 10, 15, 20
Processing time (min)	120
Temperature (K)	293.15 ± 1
Workpiece	Quartz glass (JGS1)
Size of workpiece	Φ20 mm × 1 mm
Original *R*_a_ of workpiece (nm)	2

## Data Availability

All data included in this study are available upon request by contact with the corresponding author.
